# Monitoring of the growth of *Erythrophleum fordii* and analysis of the effect of phosphorus application based on binocular vision

**DOI:** 10.3389/fpls.2026.1842583

**Published:** 2026-07-15

**Authors:** Peng Wang, Hongrong Wang, Yang Zhao, Chengde Wang, Huiyuan Cui, Xiangzhi Li, Shipeng Hou

**Affiliations:** 1 School of Forestry Engineering, Shandong Agriculture and Engineering University, Jinan, China; 2 Sichuan Forestry and Grassland Survey and Planning Institute, Chengdu, China; 3 School of Aeronautics and Astronautics, Xihua University, Chengdu, China

**Keywords:** binocular vision, three-dimensional reconstruction, *Erythrophleum fordii*, growth monitoring, phosphorus application level

## Abstract

Tree growth monitoring provides a basis for understanding tree nutritional needs, and formulating and optimizing scientific fertilization strategies, and is essential for realizing precision forestry. Computer vision can be used to reconstruct the three-dimensional geometric structure of trees from two-dimensional images, which is highly beneficial for managing and planning tree growth. Based on a binocular vision system, this study proposes an approach for monitoring the growth of *Erythrophleum fordii* and analyzing the effects of phosphorus application. The MicaSense RedEdge TM^3^ multispectral camera was used to obtain images of *E. fordii*, and the initial parameters of the camera were obtained through the Zhang Zhengyou calibration method (ZCM) and optimized using the bundle adjustment method (BA). A multi-directional semi-global stereo matching (MD-SGBM) algorithm is proposed and applied to obtain disparity information, and *E. fordii* is reconstructed based on the triangulation principle. The DBSCAN algorithm and principal component analysis (PCA) were used to denoise and correct the direction of the acquired three-dimensional point cloud, and then extract the tree height, crown width, and ground diameter growth. On this basis, a one-way analysis of variance was used to compare the growth differences of *E. fordii* under different phosphorus application levels. The results show that the combination of the beam adjustment method and Zhang Zhengyou calibration method can significantly reduce the camera reprojection error and improve the camera calibration accuracy. Compared with the actual measured values, the MAE of tree height, crown width, and ground diameter growth measured by the system were 1.315 cm, 2.128 cm, and 0.453 mm, respectively, and the RMSE were 1.561 cm, 2.520 cm, and 0.575 mm, respectively. In this experiment, low, medium, and high phosphorus treatments (5, 10, and 20 g/tree, respectively) did not significantly promote the height growth of *E. fordii*. Medium phosphorus treatment (10 g/tree) significantly promoted crown width and ground diameter growth, while under high phosphorus treatment (20 g/tree), the growth rates of both crown width and ground diameter began to decrease.

## 1 Introduction

*Erythrophleum fordii* is a tree species in the genus *Erythrophleum* of the Fabaceae family, primarily distributed in the southern coastal provinces of China, such as Guangdong, Guangxi, and Fujian, as well as in northern Vietnam. The economic value of *E. fordii* is extremely high. Its wood is hard, has a natural texture, and is highly resistant to corrosion. It is widely used in construction, furniture, and handicrafts (Zhao et al., 2015). In addition, *E. fordii* has important ecological value. It can form a symbiotic relationship with rhizobia to carry out biological nitrogen fixation, improve the nutritional status of the surrounding soil, and promote the stability and health of the ecosystem (Scheublin et al., 2004). However, with global climate change and intensified human activities, the number of wild *E. fordii* is sharply decreasing, and it has been listed in the Red List of Endangered Species of the International Union for Conservation of Nature (IUCN). To restore the population of *E. fordii* and meet market demand, people have begun to cultivate and manage the species. In this process, scientific fertilization is one of the key measures to increase the growth rate of *E. fordii* and ensure wood quality, with growth monitoring being the foundation for effective fertilization (Ouimet and Moore, 2015; Vacek et al., 2019). However, traditional growth monitoring mainly relies on manual measurements, which are time-consuming and labor-intensive. Moreover, maintaining measurement consistency and accuracy becomes challenging when monitoring large numbers of trees, limiting its applicability for large-scale, precise, and efficient growth assessment. This makes it difficult for operators to accurately assess the growth status of *E. fordii* and implement effective fertilization management and optimization (Wang et al., 2019; Jurjević et al., 2020).

With the development of computer vision and remote sensing technology, various sensing methods, such as LiDAR (Véga et al., 2014; Mahmud et al., 2021; Gülci et al., 2023), ultrasonic sensors (Tumbo et al., 2002; Palleja and Landers, 2015), and stereo vision technology (Bayati et al., 2021; Yang et al., 2022), have provided innovative solutions for determining forest growth parameters. Among them, stereo vision technology has significant advantages in terms of portability and hardware cost. Liang et al. (2016) pointed out that although the hardware cost of LiDAR is decreasing, the price range of 30,000 to 80,000 euros is still unaffordable for forestry workers. Moreover, subsequent data processing and algorithms incur additional hidden costs. Ultrasonic sensors are easily affected by environmental factors, have a limited measurement range, and exhibit poor stability (Wang et al., 2019). In contrast, stereo vision technology can reverse-engineer the three-dimensional geometric structure of trees from inexpensive and readily available two-dimensional images, enabling rapid tree reconstruction and the measurement of growth parameters.

Stereo vision technology can be divided into monocular, binocular, and multi-camera vision according to the number of cameras used and the viewing angle (Liu et al., 2012). Monocular vision acquires images through a single camera and is easy to deploy on-site, but it has high requirements on the camera’s motion trajectory and depth estimation algorithm. Therefore, it is still very challenging to use this method to obtain the three-dimensional spatial information of plants with rich textures (Zhao et al., 2022). Binocular and multi-camera vision solve this problem well. The depth of the pixel point can be estimated by calculating the disparity information of the image pair. However, multi-camera vision is inefficient and not suitable for large-scale continuous measurement. Therefore, binocular vision technology, which strikes a good balance between efficiency and accuracy, has become an ideal choice for obtaining three-dimensional spatial information about plants (Lu et al., 2020; Ma et al., 2024). A study used two webcams and a laptop to build a portable binocular stereo-vision system to reconstruct six greenhouse plants, providing an accurate measurement solution for obtaining the true size of the plants (Li et al., 2017). Another study used two visible light cameras to build a binocular vision system, measured tree characterization, and analyzed the impact of canopy shape and leaf density on the system’s measurement performance, proving the feasibility and accuracy of binocular vision technology in tree characterization measurement (Malekabadi et al., 2019). Some studies transform the spatial position of the binocular camera, obtain plant images from multiple perspectives, apply motion recovery structure (SFM) technology to build a three-dimensional model of the plant, and extract phenotypic parameters such as plant height, canopy width, and stem diameter (Ni et al., 2016; Peng et al., 2021). In addition, the canopy volume extraction system of fruit trees based on binocular vision also demonstrated good measurement accuracy, providing reliable technical support for the accurate acquisition of canopy information and the determination of pesticide spraying amount during orchard operations (Zhang et al., 2023). As the research deepens, the combination of deep learning methods and binocular vision technology has brought breakthroughs. This fusion utilizes the powerful pattern recognition capabilities of deep learning and the spatial positioning advantages of binocular vision, making it possible to accurately segment and reconstruct single plants in complex backgrounds (Niknejad et al., 2023).

Although research on the application of binocular vision technology in the field of agricultural and forestry measurement has been ongoing, it has mostly focused on the precise collection of single-temporal and spatial plant parameters. Although there are also studies using this technology to obtain multi-temporal and spatial characteristic data of crops such as lettuce, wheat, and soybeans to detect crop growth (Yeh et al., 2014; Duan et al., 2016; Ma et al., 2024), there have been no reports on the use of binocular vision technology to assess the impact of nutrients on tree growth. As one of the three major nutrients for tree growth, phosphorus plays an essential role in promoting root development, energy conversion, and enhancing tree disease resistance (Santiago et al., 2012). Despite this, there is a relative lack of analysis on the effects of phosphorus application on valuable tree species such as *E. fordii*, and no studies have explored the use of binocular vision technology to monitor the growth of *E. fordii* under different phosphorus application levels. Based on these considerations, this study constructed a binocular vision system to measure tree height, crown width, and ground diameter growth of *E. fordii* under varying phosphorus application levels, to investigate the specific effects of phosphorus on its growth. The aim is to provide an efficient and accurate growth monitoring method and fertilization decision framework, offering reliable technical support for the precise cultivation and management of valuable tree species such as *E. fordii*.

## 2 Materials and methods

### 2.1 Experimental design

**Figure 1:**
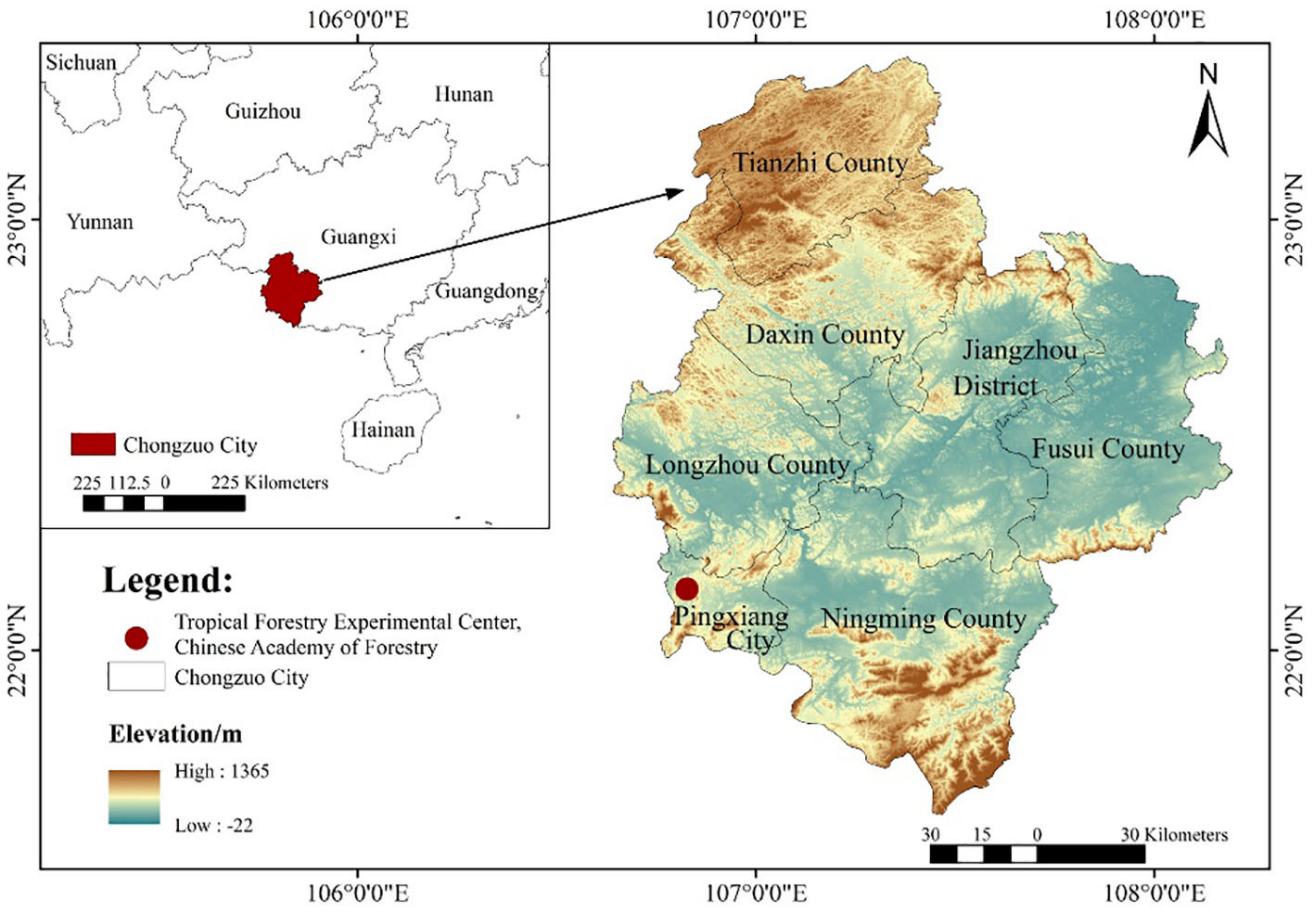
Location of the study area.

The research site is located at the nursery base of the Tropical Forestry Experimental Center of the Chinese Academy of Forestry. The specific location is shown in **Figure 1**. In this study, *E. fordii* was propagated from seeds. After two years of growth, 64 robust individuals with similar growth conditions were selected and transplanted into a set of uniform-sized pots made of the same material for cultivation. Each pot contained 50 kg of red soil, and the nutrient content of the soil is shown in **Table 1**. The pots were spaced 1 meter apart to ensure that each *E. fordii* received adequate sunlight. After a 15-day acclimatization period, a gradient phosphorus application experiment was initiated using single superphosphate (SSP) as the nutrient source. The 64 trees were divided into four groups, with the following phosphorus application rates: control group (CK, 0 g/tree), low-phosphorus group (P1, 5 g/tree), medium-phosphorus group (P2, 10 g/tree), and high-phosphorus group (P3, 20 g/tree). The experiment lasted for 4 months (from March 2024 to July 2024), with fertilization applied once a month. To maintain consistency in experimental conditions, water supply, weed control, and pest management were kept identical across all groups throughout the experiment.

**Table 1:** Nutrient content of red soil used in the research.

pH	Organic matter (g/kg)	Total nitrogen (g/kg)	Total phosphorus (g/kg)	Total potassium (g/kg)
5.99	14.95	0.91	0.66	0.63

### 2.2 Image acquisition and data collection

**Figure 2:**
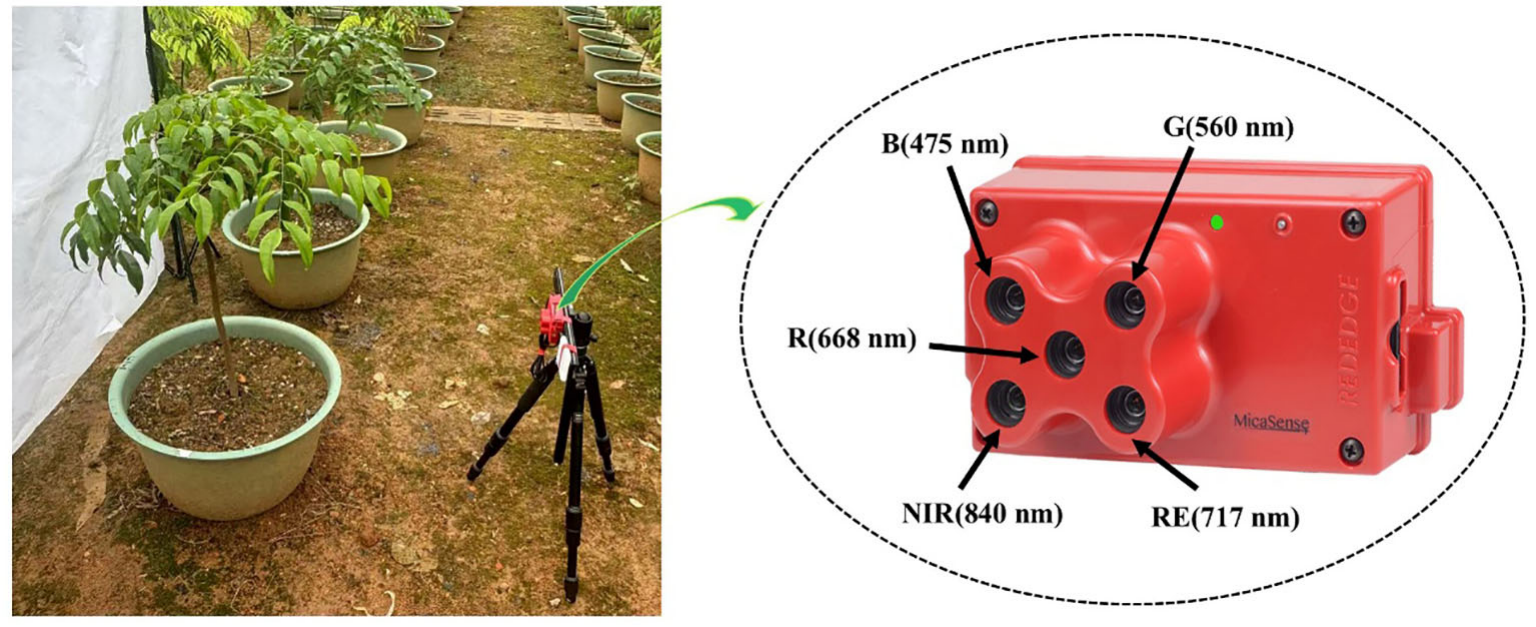
Image acquisition method and acquisition device.

In this study, the MicaSense RedEdge™ 3 multispectral sensor (hereinafter referred to as Mica) was used to capture side images of *E. fordii*. The Mica sensor is equipped with five independent and fixed spectral cameras, which allow for simultaneous collection of images in the blue (B), green (G), red (R), near-infrared (NIR), and red edge (RE) bands. Multispectral images of 64 *E. fordii* trees were collected from both the east-west and north-south directions before the experiment (March 2, 2024) and after the experiment (June 2, 2024), respectively, with an image resolution of 1280×960 pixels. During image acquisition, the Mica camera was connected to a smartphone via WIFI and mounted on a tripod. The camera’s view was monitored in real-time on the smartphone, allowing for adjustment of the tripod’s height and position. The shooting distance ranged from 0.5 m to 1.5 m, and the vertical height of the camera from the ground was between 0.5 m and 1 m, ensuring complete acquisition of the multispectral image for each *E. fordii*. The image acquisition setup is shown in **Figure 2**.

To verify the measurement accuracy of the binocular vision system, we first obtained the baseline data of the growth of the *E. fordii* by manual measurement. After each image acquisition, five experimenters used a 0.01cm scale to measure the tree height and crown width (east-west and north-south directions) of each *E. fordii* and used a vernier caliper with a scale of 0.01mm to measure the ground diameter of each *E. fordii*. After the measurement was completed, the maximum and minimum values ​​were removed, and the average of the remaining measurement values ​​was taken as the final measurement result. By calculating the difference between the two measurement results, the tree height, crown width (average of east-west and north-south crown widths), and ground diameter growth of 64 *E. fordii* before and after the experiment were obtained.

### 2.3 Image preprocessing

High-quality image input is the foundation for achieving accurate 3D reconstruction. To this end, we preprocessed the acquired raw images of *E. fordii* through logarithmic transformation, Contrast Limited Adaptive Histogram Equalization (CLAHE), and Gaussian filtering. First, the images were normalized, and a logarithmic transformation was applied according to Equation 1. Then, the images were divided into 16×16 regions (a total of 256 image blocks), and each block was individually equalized. Finally, the image blocks were merged into a complete image, with bilinear interpolation applied to smooth the edge areas, and Gaussian filtering was used for denoising.

(1) $$I_{c}=I_{o}^{\gamma }$$

Where *I_o_* is the pixel value of the original image, *I_c_* is the pixel value of the gamma-corrected image, and *γ* is the correction parameter.

### 2.4 Camera calibration

Camera calibration is one of the fundamental tasks in photogrammetry, and its main purpose is to obtain the camera parameters. Based on the results of camera calibration, image distortion can be corrected, and the position and orientation of the camera in three-dimensional space can be determined, thus establishing an accurate stereo-vision system. The chessboard calibration method (ZCM) proposed by Zhang (2000) is easy to operate and yields relatively accurate calibration results, making it widely used. ZCM is a planar calibration method, and the flatness of the calibration plate significantly affects the calibration results. However, ZCM assumes that the Z coordinates of all feature corners in the three-dimensional coordinate system are 0, neglecting the errors introduced by the bending or deformation of the calibration plate, which reduces calibration accuracy. Bundle adjustment (BA), also known as bundle adjustment, is a nonlinear global optimization method. Its core is to build an optimization model based on the initial camera parameters and the three-dimensional spatial coordinates of the feature points to minimize the reprojection error objective and solve the optimal camera parameters (Furukawa and Ponce, 2009).

Therefore, this study first used 12 sets of calibration plate images taken in advance and used the ZCM to calibrate each band camera in the Mica to obtain the initial values ​​of the internal and external parameters and feature corner coordinates of a single camera. To simulate the calibration error in the real environment, the Z coordinate error of the feature corner was imitated by the Gaussian function to obtain feature point coordinates that are more in line with reality. Subsequently, the bundle adjustment method was used to globally optimize the camera parameters and complete the single-target positioning. The reprojection error was used as an evaluation index to evaluate the accuracy of the single-target positioning. From the five-band cameras, the two cameras with the smallest reprojection error were selected to construct a binocular vision system. According to the results of the monocular camera, the relative spatial position relationship between the two cameras was calculated to obtain the system’s external parameters R and T. The calculation method is shown in Equation 2.

(2) R=RrRl-1T=Tr-RrRl-1Tl

Where ***R*** and ***T*** are the relative rotation matrix and translation vector of the left and right cameras, **R***_l_* and ***R****_r_* are the rotation matrices of the left and right cameras relative to the world coordinate system, and ***T****_l_* and ***T****_r_* are the translation vectors of the left and right cameras relative to the world coordinate system.

### 2.5 Image rectification and stereo matching

The task of binocular vision is to reconstruct the three-dimensional information of an object using a two-dimensional image pair, and stereo-matching is the key step to achieving this goal. Stereo matching is essentially the process of finding corresponding points in the left and right views, that is, finding the corresponding relationship between the projection pixels of the same real point in the three-dimensional space in the left and right views. The core of this process is to calculate the similarity of each pair of pixels, but due to factors such as illumination change and repeated texture, the accurate matching of corresponding points in the left and right views is full of challenges (Andersen et al., 2005). To reduce the amount of calculation and reduce the ambiguity of matching, auxiliary information can be introduced. Image correction can reduce the two-dimensional matching problem to one dimension, thereby improving the efficiency and accuracy of matching (Zhang, 1998). Therefore, before stereo matching, this study used the Bouguet algorithm to correct *E. fordii* image.

Accurate stereo-matching algorithms can more effectively match the key information of the left and right views, thereby improving the reconstruction accuracy. The semi-global block matching (SGBM) algorithm decomposes the global optimization problem into a one-dimensional solution problem and derives the optimal disparity solution by minimizing the energy function defined in each independent direction. It has shown a certain robustness in some application scenarios (Hirschmuller, 2007; Niknejad et al., 2023). The SGBM algorithm mainly includes four steps: cost calculation, cost aggregation, disparity calculation, and disparity optimization. In the SGBM algorithm, the cost calculation is only performed along the epipolar direction. When the image correction is biased, it will increase the difficulty of matching and cause pixel mismatching. To this end, we add the direction of cost calculation on the basis of the SGBM algorithm and propose a multi-directional semi-global matching (MD-SGBM) algorithm. In the MD-SGBM algorithm, the cost calculation is performed along the epipolar direction (from right to left), the vertical direction, and the diagonal directions from the lower right to the upper left and the upper right to the lower left. Each calculation takes the minimum cost in five directions and finally stores the cost information of all pixels in a three-dimensional cost matrix. After the cost calculation is completed, a dynamic programming strategy is used to establish the connection between adjacent pixels, further optimize the cost matrix, and realize cost aggregation. The calculation method is shown in Equations 3 and 4. The winner-takes-all (WTA) algorithm is used to select the disparity corresponding to the minimum cost value as the optimal disparity, and the one-dimensional quadratic curve fitting method is used to obtain the sub-pixel precision disparity. Finally, bilateral filtering is used to further optimize the disparity and complete stereo matching.

(3) $$L_{r}(p,d)=C(p,d)+\min\left\{\begin{array}{c} L_{r}(p-r,d)\\ L_{r}(p-r,d-1)+P_{1}\\ L_{r}(p-r,d+1)+P_{1}\\ {{\text{min}}_{i}L}_{r}(p-r,i)+P_{2} \end{array}\right\}-{\text{min}}_{k}L_{r}(p-r,k)$$

(4) $$S(p,d)=\underset{r}{\Sigma }L_{r}\left(p,d\right)$$

where *C*(*p*, *d*) is the initial cost of pixel *p* under disparity *d*, *L_r_*(*p*, *d*) is the aggregation cost of pixel *p* on path *r*, *S*(*p*, *d*) is the sum of the aggregation costs in all directions, *p*−*r* is the position of the previous pixel on path *r*, *P*_1_ and *P*_2_ are penalty terms for disparity changes, which are used to penalize slight disparity differences and significant disparity differences between adjacent pixels, respectively. min*_i_L_r_*(*p*−*r*, *i*) and min*_k_L_r_*(*p*−*r*, *k*) are minimization functions, which are used to find the minimum aggregation cost among all possible disparities *i* and *k* of the previous pixel *p*–*r*, respectively.

### 2.6 Point cloud processing and growth measurement of *E. fordii*

To remove the background parallax information, this study uses the K-means clustering (K-Mean) algorithm to segment the left view after stereo correction. After obtaining the binary image, it performs a dot multiplication operation with the disparity map to obtain a disparity map containing only *E. fordii*. According to the principle of triangulation, the image pixels are mapped to a three-dimensional space coordinate system *X*-*Y*-*Z* with the optical center of the left camera as the origin to generate a three-dimensional point cloud. The calculation method is shown in Equation 5.

(5) $$\{\begin{array}{c} X=\frac{b\times (p_{x}-c_{x})}{d}\\ Y=\frac{b\times (p_{y}-c_{y})}{d}\\ Z=\frac{b\times f}{d} \end{array}$$

Where b is the baseline length, *p*_x_ and *p_y_* are the *x* and *y* coordinates of the left view pixel, *c_x_* and c*_y_* are the *x* and *y* coordinates of the left camera optical center, *d* is the parallax, and *f* is the focal length of the camera after reprojection.

Since the generated point cloud contains a lot of noise and is inconsistent with the direction of the real-world coordinate system, we denoised and corrected the direction of the point cloud. The Density-Based Spatial Clustering of Applications with Noise (DBSCAN) algorithm was used to denoise the point cloud. Then, the centroid of the point cloud was calculated, and the main direction of the point cloud was determined by principal component analysis and a rotation matrix was constructed. The rotation matrix was used to adjust the direction of the point cloud so that it was aligned with the direction of the world coordinate system. Traverse the three-dimensional point cloud, calculate the vertical distance from the highest point of the tree to the tree base to determine the tree height, calculate the horizontal distance between the two outermost points of the tree crown to determine the crown width, calculate the Euclidean distance between the two outermost points of the tree base to determine the ground diameter, and calculate the difference between the two measurement results to obtain the tree height, crown width and ground diameter growth of the tree. The mean absolute error (MAE) and root mean square error (RMSE) are used as evaluation indicators to evaluate the measurement accuracy of the algorithm. The calculation method is shown in Equations 6 and 7. Based on this, we used Bland–Altman analysis to compare the differences between the binocular vision system and manual fine measurement of the growth of tree height, crown width, and diameter at ground level, and further evaluated the consistency and potential systematic bias between the binocular vision system measurement results and the manual measurement results.

(6) $$\text{MAE}=\frac{1}{n}\sum_{i=1}^{n}\left|y_{i}-{\hat{y}}_{i}\right|$$

(7) $$\text{RMSE}=\sqrt{\frac{1}{n}\sum_{i=1}^{n}{\left(y_{i}-{\hat{y}}_{i}\right)}^{2}}$$

Where *n* is the number of samples, *y_i_* is the *i*-th actual measurement value, and ${\hat{y}}_{i}$ is the *i*-th algorithm calculated value.

## 3 Results and analysis

### 3.1 Camera calibration results

**Figure 3:**
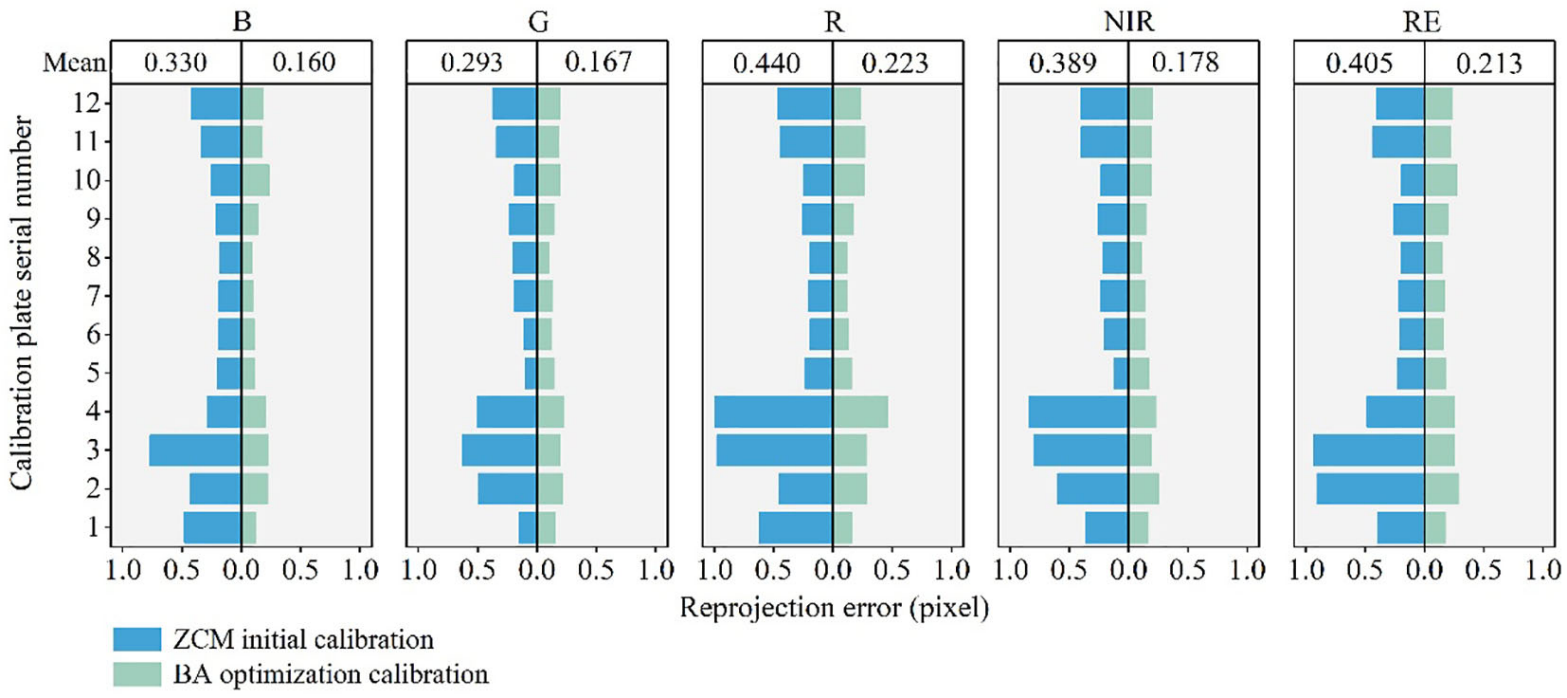
Reprojection error of camera calibration in five bands before and after BA optimization.

**Figure 3** shows the reprojection error distribution of the five-band cameras obtained by initial calibration using ZCM and optimized calibration using the BA algorithm. As shown in the figure, before applying BA optimization, the average reprojection errors of the B, G, R, NIR, and RE bands were 0.330, 0.293, 0.440, 0.389, and 0.405 pixels, respectively, indicating high reprojection errors and a wide error distribution range for each band. After BA optimization, the error distribution became more concentrated, and the average reprojection errors of each band decreased to 0.160, 0.167, 0.223, 0.178, and 0.213 pixels, respectively, representing reductions of 51.5%, 43.0%, 49.3%, 55.3%, and 47.4% compared to the optimized values. To further verify the effectiveness of BA optimization, we performed paired t-tests on the reprojection errors of each band before and after optimization. The results show that BA optimization significantly reduced the reprojection errors of all bands (*p* < 0.05). This indicates that by simulating feature corner point errors and performing BA optimization, the calibration accuracy and stability of multispectral cameras can be further improved.

Whether before or after BA optimization, the reprojection errors of the B and G band cameras are relatively smaller, indicating that the camera parameters of the two band cameras obtained by calibration are more accurate. Therefore, the G band and B band cameras are selected as the left and right cameras to build a binocular vision system. According to formula (3), the system external parameters are calculated. Combined with the results of single-target calibration, the principal point coordinates, focal length, distortion coefficients of the left and right cameras, and the rotation matrix and translation vector of the right camera relative to the left camera are finally obtained. The specific parameters are shown in **Table 2**.

**Table 2:** Calibration results of binocular vision system.

Camera parameters		Left camera	Right camera
Principal point (pixel)	X	1431	1435
	Y	1429	1435
Focal length (pixel)	X	668	667
	Y	438	473
Distortion	Radial	-0.097	-0.107
		-0.093	-0.114
	Tangential	-0.097	-0.004
		0.005	0.005
Rotation matrix	[1, 0.002, 0.001; -0.002, 1, -0.010; -0.001, 0.010,1]		
Translation vector	[-29.891, -0.013, -0.384]		

### 3.2 Disparity map and 3D point cloud

**Figure 4:**
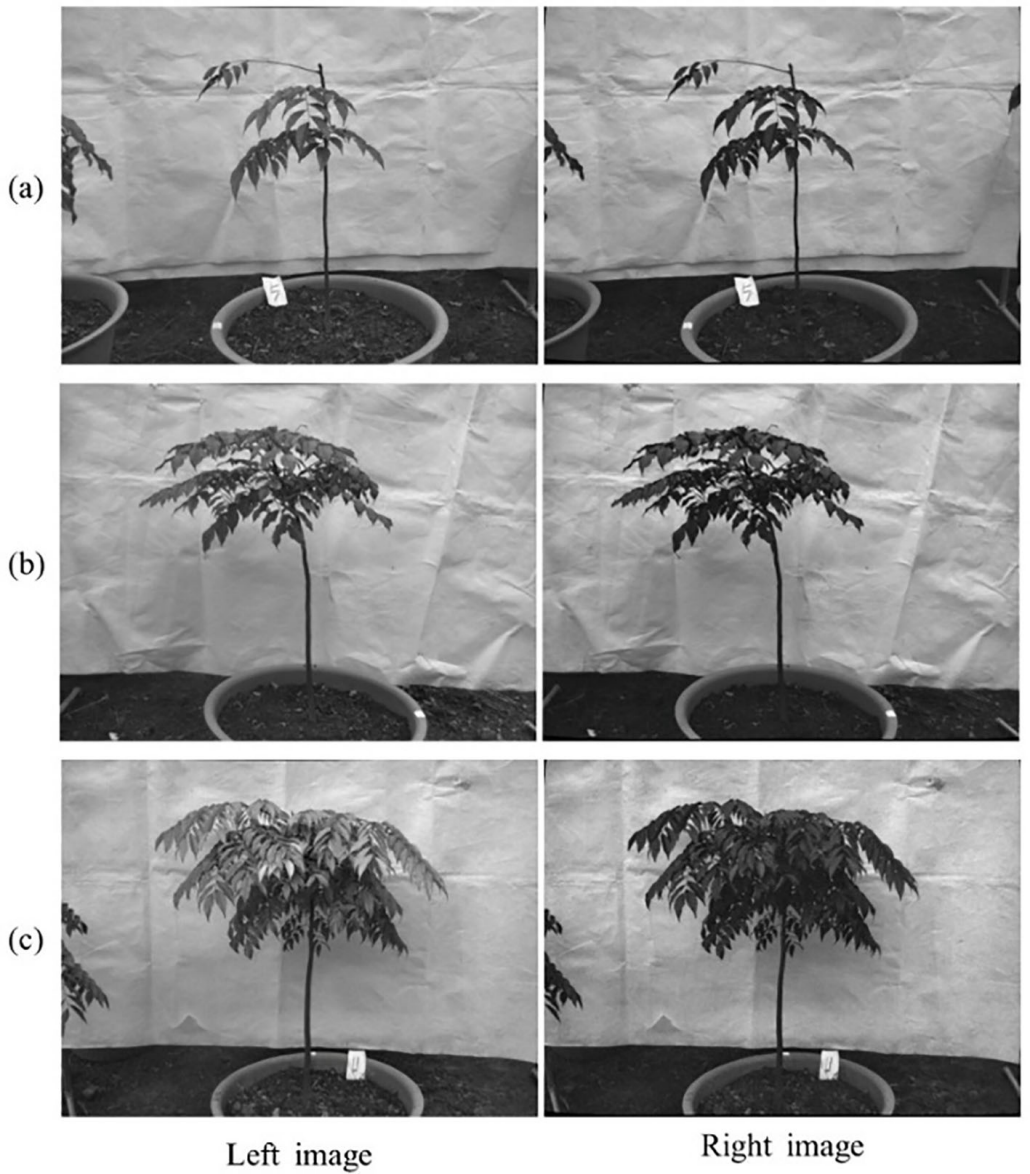
Preprocessed and corrected *Erythrophleum fordii* image pair. **(A)** Sparse density. **(B)** medium density. **(C)** dense density.

**Figure 5:**
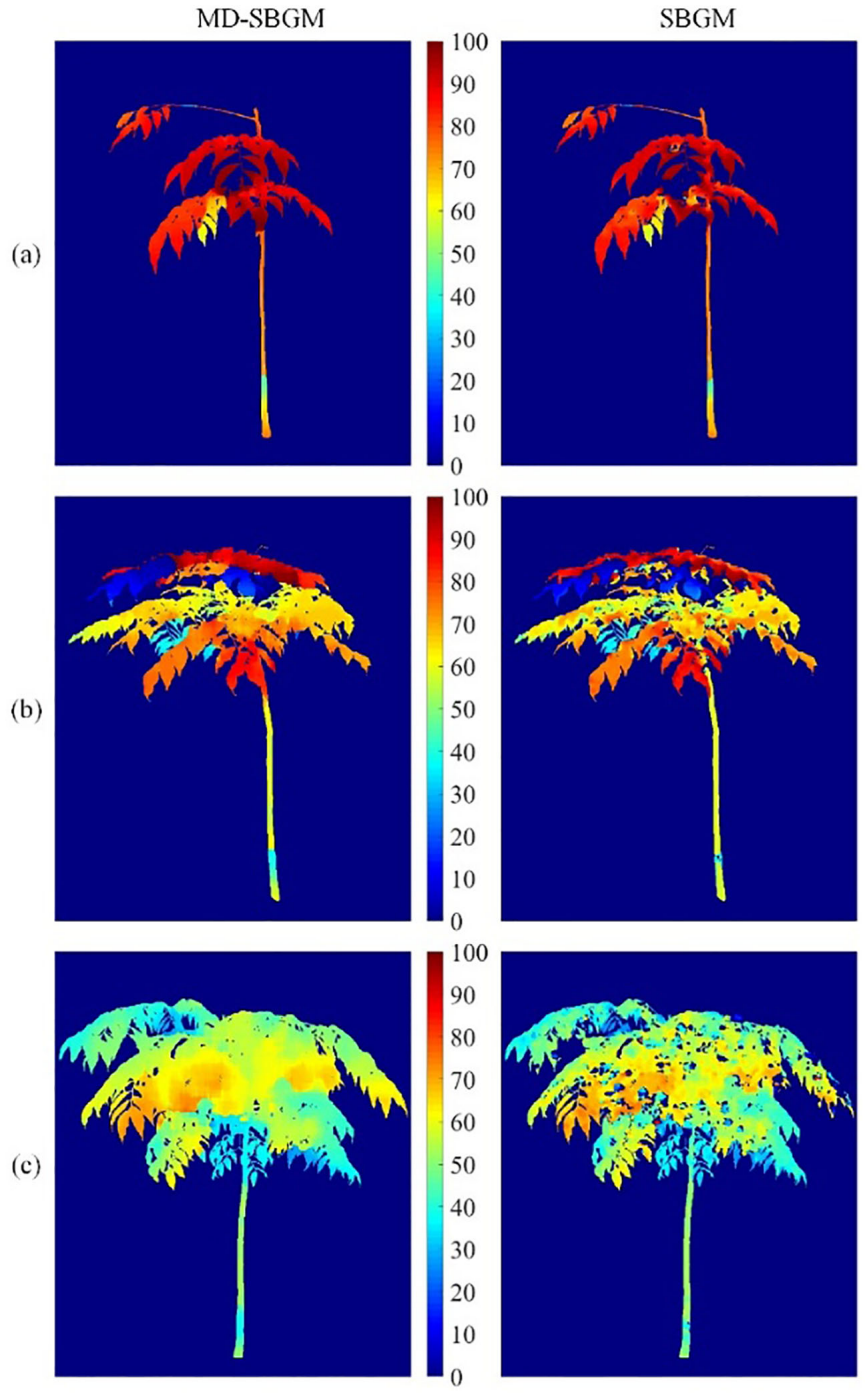
Disparity map generated by matching MD-SGBM and SGBM algorithms. **(A)** Sparse density. **(B)** medium density. **(C)** dense density.

This study compares the performance differences between the MD-SGBM algorithm and the SGBM algorithm in matching the images of *E. fordii* with different canopy densities (sparse, medium density, and dense). **Figure 4** shows the *E. fordii* image pair after preprocessing and image correction, and **Figure 5** shows the disparity map generated by the two algorithms. Two methods are used to evaluate the disparity map, one is visual subjective evaluation, and the other is objective evaluation, that is, calculating the peak signal-to-noise ratio (PSNR) and the percentage of bad matching pixels (BMP) for evaluation. The larger the PSNR and the smaller the BMP, the lower the noise level and mismatch rate of the disparity map (Scharstein and Szeliski, 2002; Malpica and Bovik, 2009). Through visual interpretation, it can be seen that for the sparse density canopy, the disparity maps generated by the MD-SGBM and SGBM algorithms do not produce obvious noise, with PSNRs of 31.40db and 29.49db respectively, but the disparity image generated by MD-SGBM is more complete, with a BMP of 1.75%, while the BMP of the disparity map matched by the SGBM algorithm is 6.80%. In the scene with medium canopy density, the disparity image generated by the MD-SGBM algorithm is relatively smooth, with a PSNR and BMP of 33.66db and 0.51% respectively, while the disparity map generated by the SGBM algorithm produces a small amount of noise, with a PSNR and BMP of 23.53db and 5.31% respectively. In the case of dense canopy, the disparity map generated by the MD-SGBM algorithm is smooth and continuous, with no obvious missing, with a PSNR and BMP of 30.72db and 0.56% respectively, while the disparity map generated by the SGBM algorithm produces a lot of noise and mismatched areas with a PSNR and BMP of 17.99db and 10.50% respectively.

**Figure 6:**
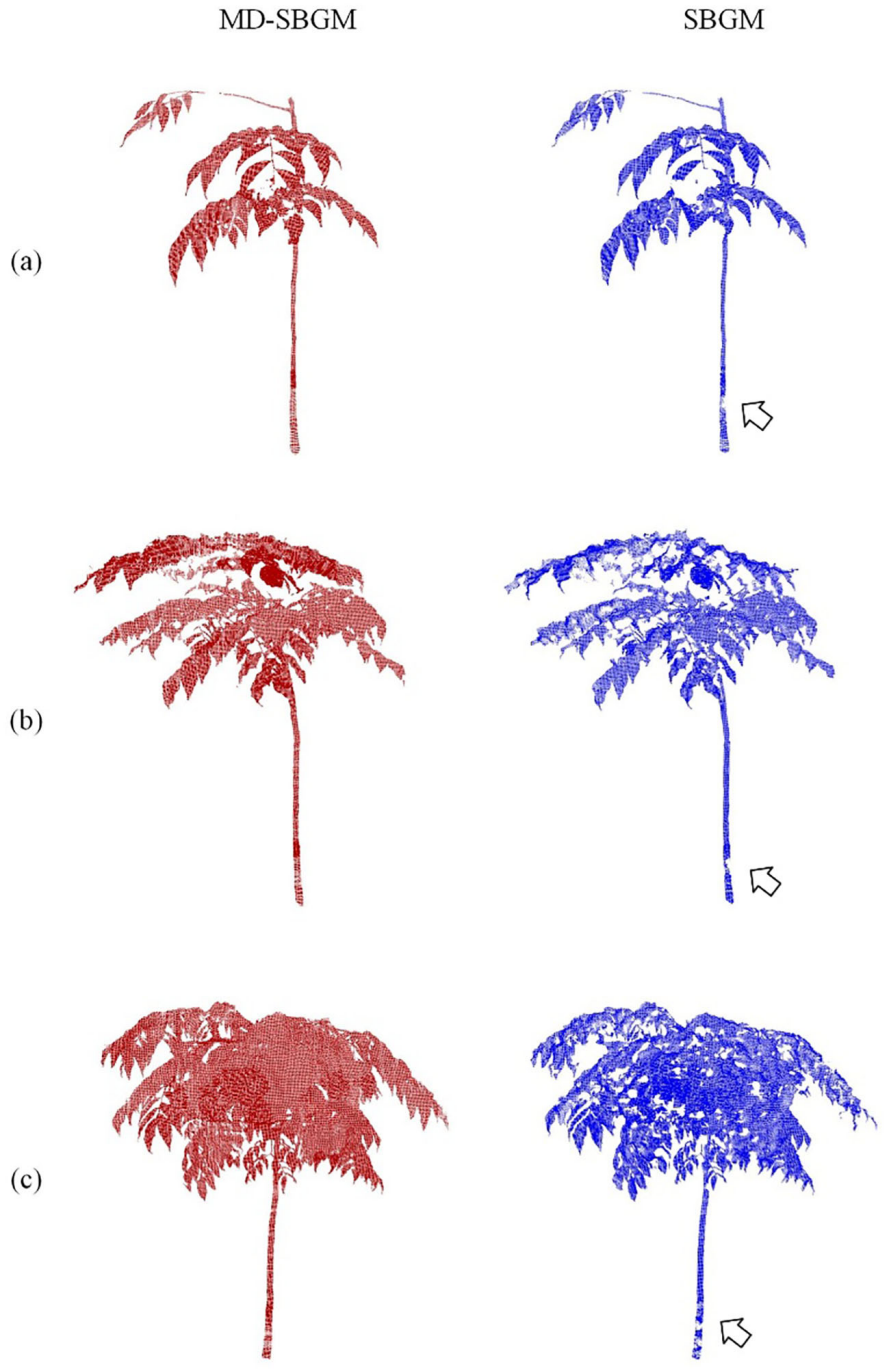
3D point cloud of *Erythrophleum fordii* generated based on MD-SGBM and SGBM algorithms. **(A)** Sparse density. **(B)** medium density. **(C)** dense density.

**Figure 6** shows the three-dimensional point cloud of *E. fordii* generated by the matching results of the MD-SGBM and SGBM algorithms. The quality of the point clouds generated by the two algorithms is comprehensively evaluated by counting the number of point clouds and detecting outliers. The total number of *E. fordii* point clouds with sparse, medium, and dense canopy densities generated by the MD-SGBM algorithm is 49281, 102412, and 185700, respectively, and the number of outliers is 1943, 3848, and 6291; the total number of point clouds with three canopy densities generated by the SGBM algorithm is 47126, 96915, and 168467, respectively, and the number of outliers is 2570, 4521, and 7988. In general, the point cloud generated by the MD-SGBM algorithm is denser than that of the SGBM algorithm, and there is no significant point cloud missing as shown by the arrow in **Figure 6**, and the number of outliers is less.

A comprehensive analysis of **Figures 5** and **6** shows that the MD-SGBM algorithm is not affected by the canopy density. The disparity map generated by matching in the three density modes is continuous and smooth, and the quality of the generated three-dimensional point cloud is high. The SGBM algorithm can achieve better-matching results in sparse scenes. However, with the increase of canopy density, the SGBM algorithm produces mismatches in some repeated texture areas, the parallax continuity is reduced, and the generated point cloud is relatively sparse and has a high noise level.

### 3.3 Determination of tree growth

#### 3.3.1 Determination of tree height growth

**Figure 7:**
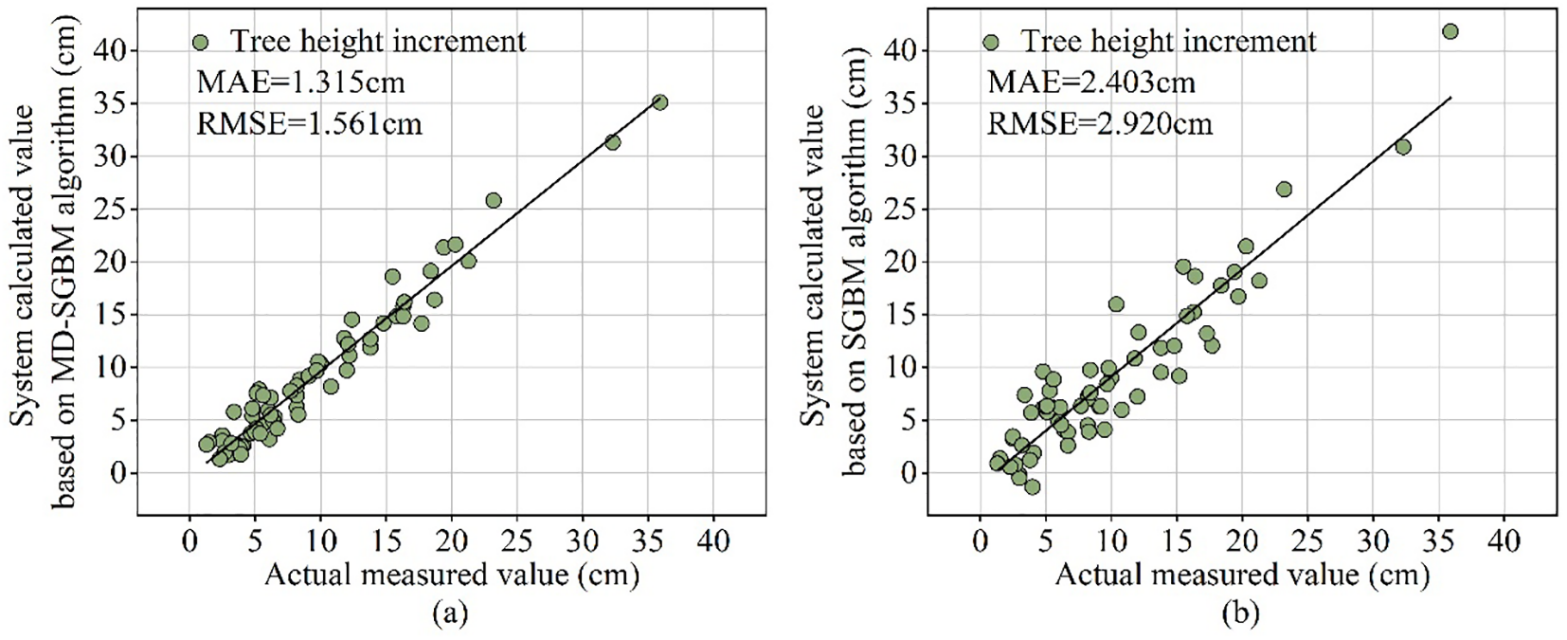
Tree height growth calculation results. **(A)** Linear correlation between the calculated value of the MD-SGBM algorithm and the measured value. **(B)** Linear correlation between the calculated value of the SGBM algorithm and the measured value.

The MD-SGBM and SGBM algorithms were used to obtain disparity values ​​and measure the tree height growth, respectively, to compare the accuracy of the two algorithms in measuring tree height growth. **Figure 7** shows the linear regression model of the tree height growth calculated by the two algorithms and the actual measured values, with *R*^2^ of 0.951 and 0.868 respectively. The tree height growth calculated by the MD-SGBM algorithm is positive, while the SGBM algorithm has negative values, indicating that compared with the MD-SGBM algorithm, the SGBM algorithm is prone to generate abnormal disparity values ​​when matching images, resulting in excessive overestimation or underestimation of the tree height. The error index further reflects the performance difference between the two algorithms. The MAE and RMSE of the system calculated value based on the MD-SGBM algorithm and the actual measured value are 1.315cm and 1.561cm respectively, while the MAE and RMSE of the SGBM algorithm calculated value and the actual measured value are 2.403cm and 2.920cm respectively. Compared with the SGBM algorithm, the MD-SGBM algorithm is more stable and accurate in measuring tree height growth.

**Figure 8:**
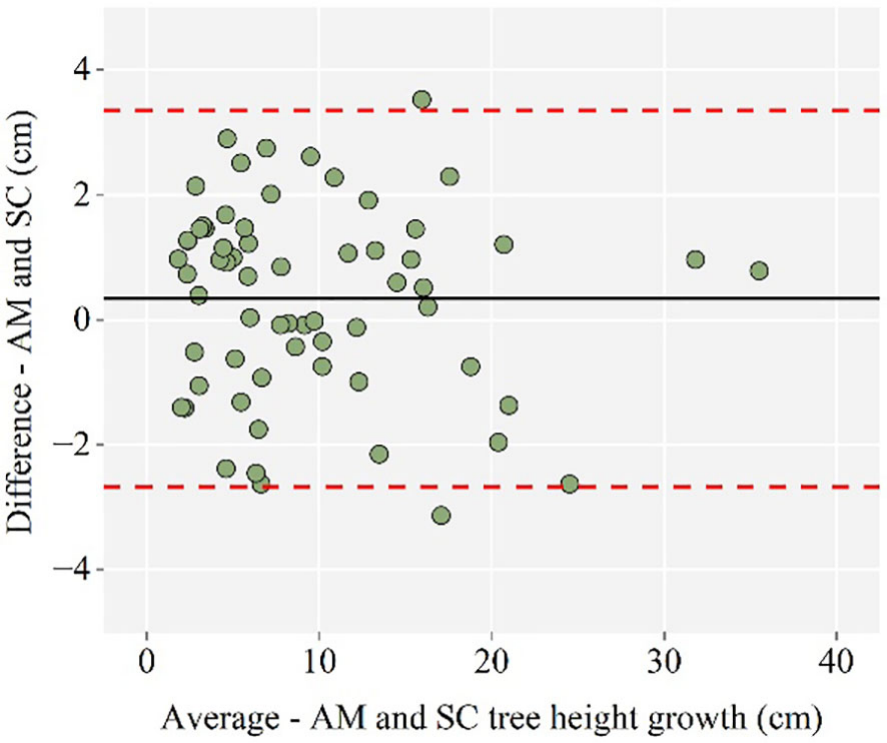
Bland-Altman analysis on system calculated (SC) tree height growth against actual measurements (AM).

Bland-Altman analysis was used to further test the accuracy of the MD-SGBM algorithm in measuring tree height growth, and the results are shown in **Figure 8**. **Figure 8** shows that 93% of the data points are within the consistency limit, and the upper limit of the consistency limit between the system-calculated (SC) value and the actual measured (AM) value is 3.348cm, the lower limit is −2.672cm and the mean difference is 0.338cm, which shows that the difference between most of the system calculated values and the measured values is within a reasonable range and meets the consistency requirements. In addition, the results of the t-test on the mean difference show that the t-value is 1.761 and the *p*-value is 0.083, indicating that the mean difference between the system-calculated value and the measured value is not statistically significant, and there is no systematic deviation between the two.

#### 3.3.2 Determination of crown growth

**Figure 9:**
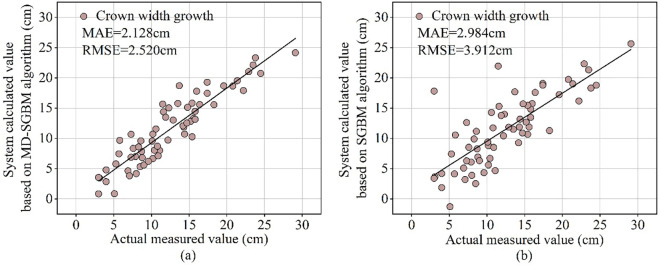
Calculation results of crown width growth. **(A)** Linear correlation between the calculated value of MD-SGBM algorithm and the measured value. **(B)** Linear correlation between the calculated value of SGBM algorithm and the measured value.

The system-calculated results based on the MD-SGBM and SGBM algorithms were evaluated according to the actual measured crown width growth. As shown in **Figure 9**, the *R*^2^ of the linear regression model between the system-calculated ​​based on the two algorithms and the measured values ​​were 0.844 and 0.624, respectively. The system-calculated values ​​based on the MD-SGBM algorithm were all positive values, with an MAE of 2.128 cm and an RMSE of 2.520 cm compared with the measured values, while negative values ​​appeared in the system-calculated values ​​based on the SGBM algorithm, with an MAE of 2.984 cm and an RMSE of 3.912 cm compared with the measured values. The above results show that in the calculation of crown width growth, the MD-SGBM algorithm performs more stably and has higher measurement accuracy.

**Figure 10:**
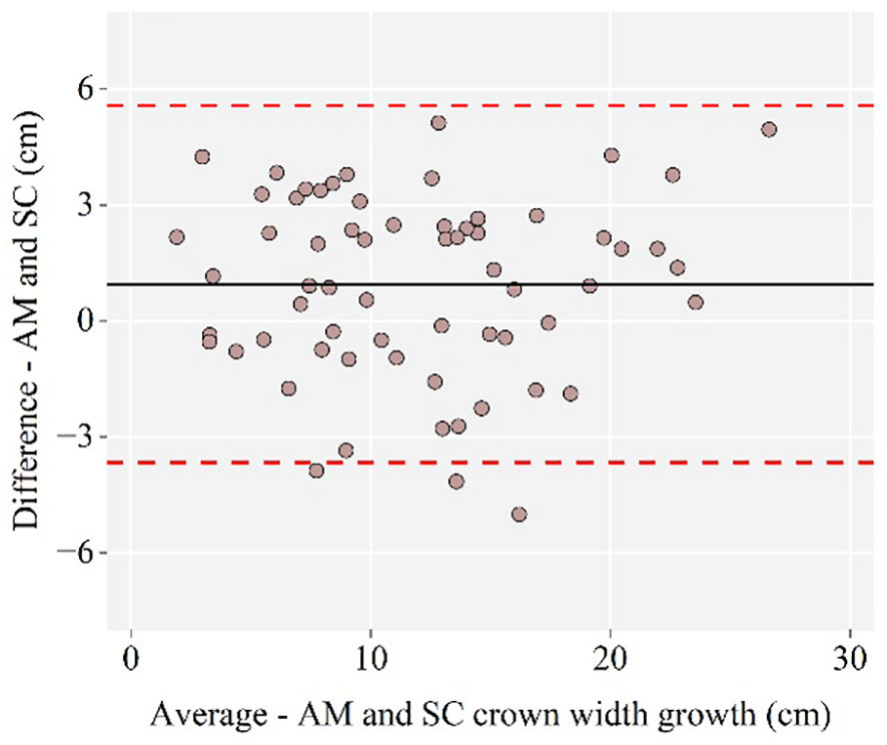
Bland-Altman analysis on system calculated (SC) crown width growth against actual measurements (AM).

**Figure 10** shows the Bland-Altman analysis results of the crown width growth of the system-calculated (SC) value and the actual measurement (AM) based on the MD-SGBM algorithm. The upper and lower limits of the consistency limit are 5.560cm and −3.668cm, respectively, and the mean difference between the system-calculated value and the measured value is 0.946cm. The difference was further analyzed by t-test, and the results showed that the t-value was 3.214 and the p-value was 0.002 (*p* < 0.05), which indicates that there is a non-zero deviation between the system-calculated results and the measured results, and the system overestimates the crown width growth.

#### 3.3.3 Determination of ground diameter growth

**Figure 11:**
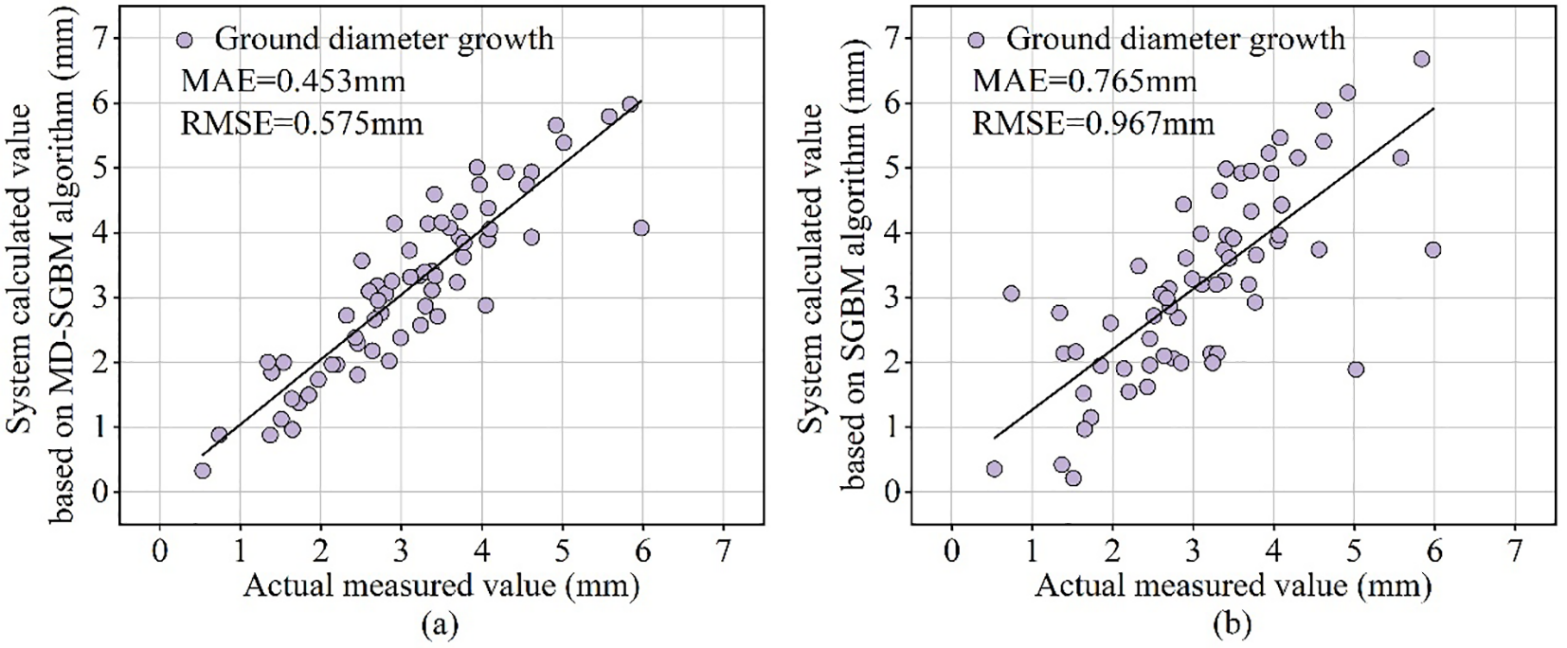
Calculation results of ground diameter growth. **(A)** Linear correlation between the calculated value and the measured value by MD-SGBM algorithm. **(B)** Linear correlation between the calculated value and the measured value by SGBM algorithm.

**Figure 11** shows the linear regression relationship between the ground diameter growth calculated by stereo matching using the MD-SGBM algorithm and the SGBM algorithm and the actual measured value. The R^2^ of the linear regression model between the MD-SGBM algorithm calculated value and the manual measured value is 0.804, while the *R*^2^ of the linear regression model between the SGBM algorithm calculated value and the manual measured value is only 0.561. At the same time, most of the data points in **Figure 11A** are evenly distributed on both sides of the fitting line, while the data points in **Figure 11B** are relatively scattered. Analysis of the error index shows that the MAE and RMSE of the system-calculated value based on the MD-SGBM algorithm and the measured value are 0.453mm and 0.575mm, respectively, while the MAE and RMSE of the system-calculated value based on the SGBM algorithm and the measured value are 0.765mm and 0.967mm, respectively. This shows that compared with the SGBM algorithm, the linear relationship between the ground diameter growth calculated by the MD-SGBM algorithm and the measured value is stronger, and the measurement accuracy is higher.

**Figure 12:**
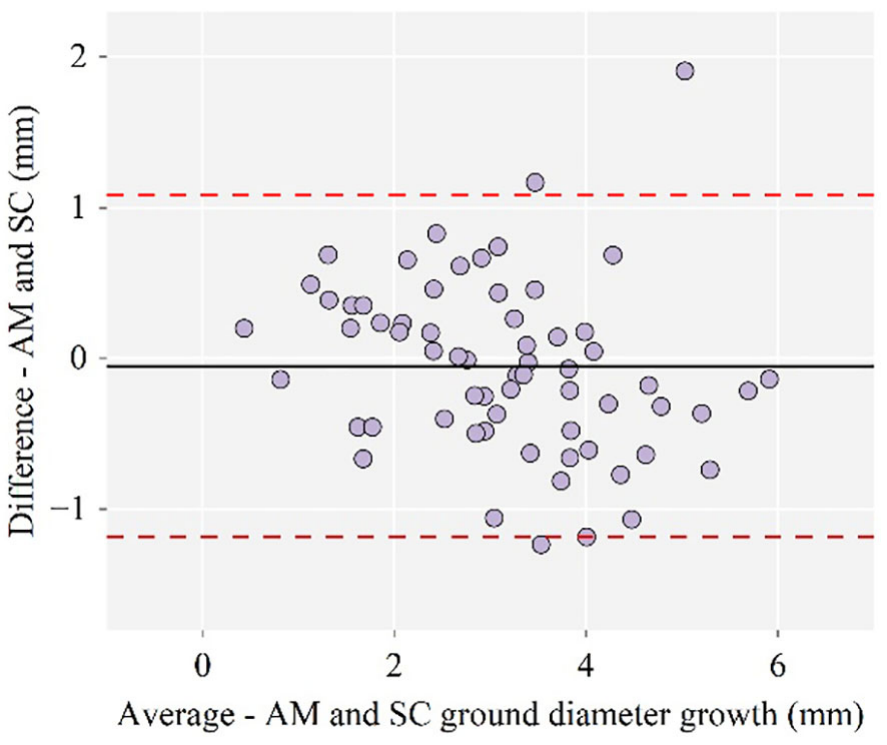
Bland-Altman analysis on system calculated (SC) ground diameter growth against actual measurements (AM).

**Figure 12** shows the Bland-Altman analysis results of the actual measurement (AM) and the system (based on the MD-SGBM algorithm) measured (SC) ground diameter growth. The data points in the figure are evenly distributed around the zero difference line, and 93% of the data points are within the consistency limit. The upper limit of the consistency limit is 1.082mm, the upper limit is −1.182mm, and the difference mean is −0.04mm, indicating that the system measurement method and the actual measurement method have strong consistency. The *p*-value of the slope of the measured difference value and the average value is 0.051, which is slightly higher than the significance level of 0.05, indicating that there is no significant linear relationship between the difference value and the mean, but there is a marginal trend. The t-test results of the difference mean show that the t-value is −1.060 and the *p*-value is 0.293, indicating that there is no significant deviation between the system measurement value and the manual measurement value, and the difference is only manifested as a random error.

### 3.4 Analysis of phosphorus application effect

**Figure 13:**
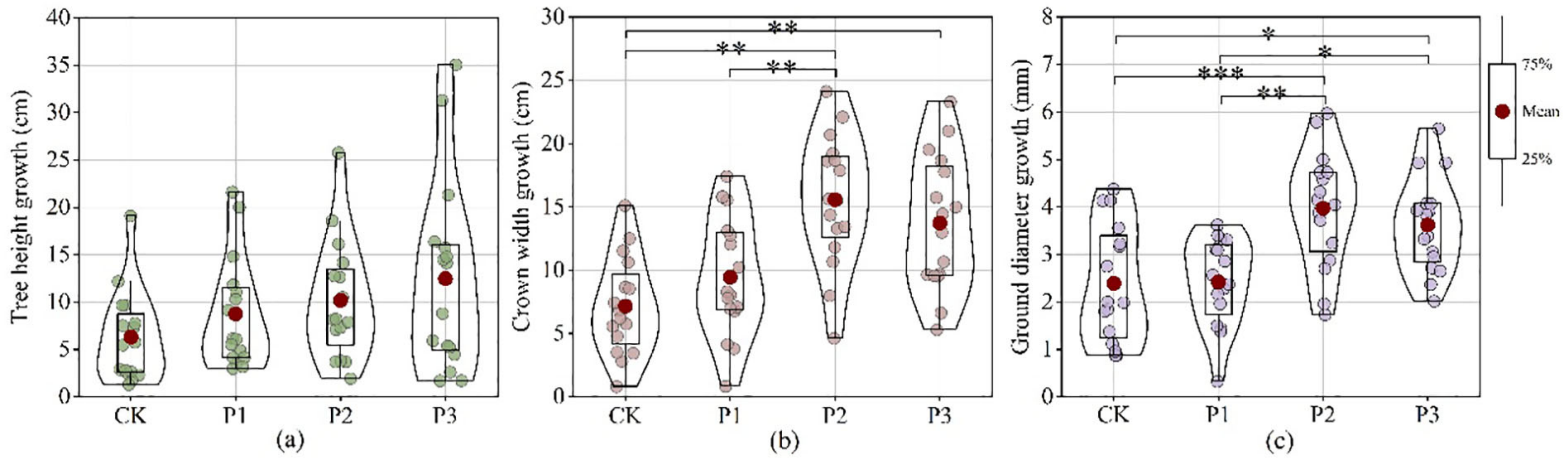
Differences in growth of *Erythrophleum fordii* under different phosphorus application levels. **(A)** Tree height. **(B)** Crown growth. **(C) **Ground diameter.

A one-factor variance analysis was performed based on the system-measured *E. fordii* growth, and the results are shown in **Figure 13**. **Figure 13A** shows that the average tree height growth of the P1, P2, and P3 groups was higher than that of the CK group, and as the level of phosphorus application increased, the tree height growth showed an upward trend. It is worth noting that the standard deviation of the P3 group reached 10.118cm, indicating that there is large variability in this group. The results of the one-way analysis of variance showed that the partial η² was 0.096, indicating that phosphorus concentration contributed 9.6% to the variation of tree height growth. Phosphorus application affected the growth of tree height to a certain extent. However, at the 0.05 level, there was no significant difference in tree height growth among each group.

**Figure 13B** shows that as the level of phosphorus application increases, the average crown growth first increases and then decreases. The average growth of each group is P2>P3>P1>CK. The results of the one-factor analysis of variance showed that the partial η² was 0.340, indicating that the variation contribution of phosphorus concentration to crown width growth was 34.0% and phosphorus application significantly affected the growth of crown width. Among them, the P2 group increased significantly by 117.6% and 45.4% respectively compared with the CK and P1 groups (*p* < 0.01). The P3 group increased significantly by 91.9% compared with the CK group (*p* < 0.01). CK and P1, P2 and P3, There was no significant difference in crown width growth between P1 and P3 groups.

**Figure 13C** shows that as the level of phosphorus application increases, the average growth amount of ground diameter first increases and then decreases. The average growth amount of ground diameter in each group is P2>P3>P1>CK. The results of the single-factor analysis of variance showed that the partial η² was 0.302, indicating that phosphorus concentration contributed 30.2% to the variation of ground diameter growth, and phosphorus application significantly affected the growth of ground diameter. Among them, the P2 group increased significantly by 66.0% (*p* < 0.001) compared with the CK group and by 63.8% (*p* < 0.01) compared with the P1 group. The P3 group increased significantly by 51.3% and 49.3% compared with the CK and P1 groups, respectively (*p* < 0.05). There was no significant difference between the P1 group and the CK group, and between the P2 group and the other groups.

## 4 Discussion

### 4.1 Advantages of using binocular vision technology to monitor tree growth

Traditional forest monitoring methods are mainly based on manual measurement, which is not only time-consuming and laborious, but also the sparse data obtained makes it difficult for operators to fully understand the growth mechanism of trees. To this end, this study proposes a method for monitoring the growth of trees based on binocular vision technology. Using the method of this study, the three-dimensional structure of trees can be quickly obtained through the captured image pairs, and then the tree growth factor can be measured, avoiding the cumbersome data collection process of the manual measurement method and the inaccurate expression of morphological features caused by the two-dimensional image method due to the inability to obtain depth information. Although other sensing methods can also solve these problems, such as using aerial light detection and LiDAR ranging systems to accurately obtain the three-dimensional geometric structure and specific morphology of trees (Malhi et al., 2018; Sangjan and Sankaran, 2021). However, compared with our method, the cost of these technologies is undoubtedly expensive. At the same time, because the original point cloud model obtained contains complex background and noise, a lot of effort must be spent to obtain a higher level of detail (Niknejad et al., 2023). The application of binocular vision technology can first obtain a parallax image without background, and then generate a point cloud, which can greatly shorten the post-processing steps of the point cloud while ensuring accuracy.

In addition, the results produced by our method are more accurate than the existing remote sensing methods for tree measurement. Yin et al. (2024) used LiDAR sensors to monitor the growth of mangroves. The deviation from the tree height measured on site was between −9 and +11 cm, while the MAE of the height growth of the loblolly tree measured by us was 1.315 cm. Kukkonen et al. (2022) estimated the trunk diameter based on the LiDAR system. The RMSE with the measured value was 3.9 mm, and the RMSE of the ground diameter growth we measured was 0.575 mm. Moorthy et al. (2011) used a ground laser scanner to obtain the characterization parameters of 24 olive trees. The RMSE of the crown width measured by the system was 14 cm. In comparison, our results are more accurate, and the RMSE of the measured crown width growth is only 2.128 cm. It is worth noting that in this study, there was no significant deviation between the tree height and ground diameter growth measured by the system and the measured values, while the system overestimated the crown growth. This is because the crown structure is relatively complex and there are too many repeated textures, which leads to deviations in parallax calculations. At the same time, the existence of occlusion and wind factors further reduces the accuracy of crown width measurement.

### 4.2 Key technologies for binocular accurate tree measurement

The premise of achieving binocular precision measurement is to accurately obtain camera parameters. However, due to factors such as lens distortion, uneven response of camera sensors, and changes in illumination, traditional calibration methods often fail to meet the requirements of high-precision calibration. To this end, this study uses the Zhang Zhengyou calibration method (ZCM) for preliminary camera calibration, and then applies the bundle adjustment method to eliminate nonlinear errors, so that the average reprojection error of the binocular imaging system is reduced to less than 0.2 pixels. This method significantly improves the accuracy of camera calibration and provides a reliable parameter basis for subsequent three-dimensional reconstruction and depth estimation. Cui et al. (2014) introduced epipolar constraints and constant distance constraints on the basis of ZCM, which effectively reduced the systematic error in camera calibration. Yin et al. (2012) proposed a camera calibration method based on multi-constraint optimization, which significantly reduced the camera reprojection error compared to ZCM. However, these methods greatly increase the calibration time. In comparison, our method is simpler and can ensure calibration efficiency while improving accuracy.

In addition, an accurate stereo-matching algorithm is also the key to achieving binocular precision measurement. In the study of greenhouse plant reconstruction, Li et al. (2017) applied the adaptive weighting method to improve the traditional ADCensus algorithm to achieve stereo matching. The results showed that the pixel mismatch rate was significantly reduced, and the single-point measurement error was less than 5 mm. Malekabadi et al. (2019) used the ABGM algorithm to obtain tree disparity maps and measure tree representations. The measurement accuracy of crown width was -1%~3% and the measurement error of trunk diameter was -8%~5%. Fu et al. (2023) measured the DBH of 48 standing trees by calculating disparity using the SGBM algorithm. The RMSR and MAE of the DBH extracted by the algorithm and the measured DBH were 3.13 cm and 3.11 cm, respectively. Niknejad et al. (2023) applied the SGBM algorithm to match pine images in the constructed binocular vision system. After generating the point cloud, the trunk diameter, branch angle, and diameter were extracted, and the RMSE of 0.055m, 5.0˚, and 5.6 mm were achieved, respectively. In this study, the MD-SGBM algorithm we proposed adds the direction of cost calculation on the basis of the traditional SGBM algorithm, making the obtained disparity more accurate, thereby achieving an accurate measurement of the growth of the eucalyptus. Although this improvement increases the computational burden to a certain extent, the accuracy gain it brings is worthwhile in high-precision agricultural and forestry measurement tasks.

### 4.3 Limitations and future directions

Accurate segmentation of the disparity map can ensure that only three-dimensional point clouds containing tree information are generated. When the segmentation accuracy is low, the generated point clouds will contain more noise points, thereby reducing the accuracy of the measurement. Although this study uses the K-Means algorithm to better distinguish between trees and backgrounds, the algorithm does not have self-adaptability, which means that it cannot automatically adjust the segmentation strategy in different scenarios. When the K value is not selected properly, it is easy to produce segmentation errors (Dhanachandra et al., 2015; Zheng et al., 2018). Therefore, in future research, heuristic algorithms can be used to search for the optimal K value adaptively, to further improve the segmentation accuracy. Theoretically, the more disparity search directions there are, the more accurate the matching disparity will be. However, considering the matching efficiency, the MD-SGBM algorithm we proposed only adds 4 disparity search directions based on the SGBM algorithm. In future studies, the MD-SGBM algorithm will continue to be improved, and more search directions will be added while ensuring matching efficiency, to further improve the measurement accuracy of the algorithm.

In addition, in this study, we only analyzed the effect of phosphorus application level on the growth of *E. fordii*. In fact, according to the method of this study, a non-invasive and efficient fertilization decision-making framework can be constructed. Through binocular vision technology, tree growth can be quickly and accurately measured, and the most conducive fertilization method for tree growth can be determined, to formulate and adjust the fertilization strategy and promote the healthy growth of trees. It is worth noting that under the experimental conditions of this study, phosphate application had no significant effect on the height of *E. fordi*. However, considering the relatively small sample size and the short experimental duration, the lack of significance may be due to limited statistical power; the possibility of type II error cannot be ruled out. Therefore, future research will further refine the experimental design, increase the sample size and experimental duration, and consider the interactions and synergistic effects of different nutrients to determine the most favorable fertilization strategy for *E. fordii* growth, providing a reference for the precision cultivation of valuable tree species such as *E. fordii*.

It should be noted that the subjects of this study were saplings, all of which were potted plants with minimal shading, making the measurement conditions relatively controllable. However, actual forest stands have complex topography, significant light variations, and tall, dense trees with overlapping canopies, all of which reduce measurement accuracy. Nevertheless, this limitation does not preclude the application of the proposed framework. Future research could utilize UAVs equipped with binocular vision systems to acquire forest stand images from different heights and angles and reconstruct the canopy structure. Simultaneously, combining this with ground-based binocular vision systems to acquire information on the sub-canopy structure and achieving aerial-ground data fusion through point cloud registration would improve the accuracy of individual tree reconstruction and the extraction of tree measurement factors such as height, crown width, and diameter at root, further enhancing the application potential of binocular vision technology in large-scale forest resource monitoring.

## 5 Conclusion

In this study, a binocular vision system was applied to monitor the growth of *E. fordii* and evaluate the effects of phosphorus application. A binocular vision system was constructed using two single-band cameras of the Mica sensor. The initial parameters of the system were obtained using ZCM and optimized using the BA algorithm to reduce the system reprojection error to less than 0.2 pixels. A multi-directional semi-global stereo matching algorithm (MD-SGBM) was proposed and applied to calculate the disparity map of *E. fordii*. After generating a three-dimensional point cloud based on the triangulation principle, the growth of *E. fordii* height, crown width, and ground diameter was measured. The maximum RMSE between the system-calculated value and the measured value was 2.520 cm, and the minimum was 0.575 mm. Based on the system calculation results, the growth differences of *E. fordii* under different phosphorus application levels were analyzed. The results showed that under the experimental conditions, phosphorus application had no significant effect on the plant height of *E. fordii*. Low phosphorus treatment (5 g/plant) had a weak and insignificant effect on the crown width and diameter growth of *E. fordii*. Medium phosphorus treatment (10 g/plant) significantly promoted the crown width and diameter growth of *E. fordii*. However, high phosphorus treatment (20 g/plant) had an inhibitory effect, reducing the growth rate of crown width and ground diameter of *E. fordii*.

## Data Availability

The original contributions presented in the study are included in the article/supplementary material. Further inquiries can be directed to the corresponding authors.
